# Examination of Young US Adults’ Reasons for Not Seeking Mental Health Care for Depression, 2011-2019

**DOI:** 10.1001/jamanetworkopen.2022.11393

**Published:** 2022-05-10

**Authors:** Wenhua Lu, Melissa Bessaha, Miguel Muñoz-Laboy

**Affiliations:** 1 Department of Community Health and Social Medicine, School of Medicine, The City University of New York, New York; 2 School of Social Welfare, Stony Brook University, Stony Brook, New York

## Abstract

This survey study uses data from the National Survey on Drug Use and Health to examine trends and patterns in young adults’ perceived reasons for not seeking treatment for depression.

## Introduction

Compared with any other adult age group, depression is most prevalent among young adults aged 18 to 25 years in the US.^1^ Despite the increasing trajectory of depression in the past decade, young adults’ use of treatment for depression remains low.^2^ Untreated depression increases young adults’ risk for substance abuse, risky sexual behaviors, unemployment, and suicide.^3^ This study aimed to examine trends and patterns in young adults’ perceived reasons for not seeking treatment for depression.

## Methods

This study used nationally representative data from the 2011-2019 National Survey on Drug Use and Health (NSDUH) for civilian, noninstitutionalized young adults aged 18 to 25 years.^4^ Young adults with a 12-month major depressive episode (MDE) based on *Diagnostic and Statistical Manual of Mental Disorders* (Fourth Edition) criteria were asked whether they had received any mental health treatment in the past year; those who responded “no” were further surveyed about reasons why they did not seek treatment. The sociodemographic variables that were examined included young adults’ age, sex, race and ethnicity, and annual household income. The institutional review board at RTI International approved the NSDUH data collection protocol, and verbal informed consent was obtained from each participant. This study followed the American Association for Public Opinion Research (AAPOR) reporting guideline for survey studies.^5^

Bivariate logistic regression analyses were first conducted to assess time changes in annual proportions of young adults who reported specific reasons for not seeking treatment for depression (0 = no; 1 = yes); survey year was the continuous independent variable. For reasons with statistically significant time changes, interaction effects between survey year and sociodemographic variables were further examined in respective bivariate regression models. Last, a series of multivariable logistic regression analyses were conducted to examine sociodemographic differences in participants’ reported reasons for not seeking treatment for depression, controlling for survey year, participants’ MDE-related severe functional impairment, and sampling weights.

All analyses were performed using R, version 4.0.3 (R Group for Statistical Computing). For the 2011-2019 NSDUH, the annual mean weighted interview response rates for adults aged 18 to 25 years ranged between 66.4% and 80.5%. All *P* values were from 2-sided tests and results were deemed statistically significant at *P* ≤ .05.

## Results

Between 2011 and 2019, 11 186 of 21 012 young adults with a 12-month MDE did not receive any treatment, among whom 6837 (61.1%) were women, 4349 (38.9%) were men, 4412 (39.4%) were aged 18 to 21 years, 6283 (56.2%) were White, 3309 (29.6%) had an annual household income of less than $20 000, and 6363 (56.8%) had MDE-related severe functional impairment. The sociodemographic distribution was largely consistent across survey years.

In 2019, the most-reported reasons by young adults for not seeking treatment for an MDE were cost (776 of 1552 [54.7%]; weighted percentage), not knowing where to go for services (572 of 1552 [37.8%]; weighted percentage), thought they could handle the problem without treatment (525 of 1552 [30.9%]; weighted percentage), and fear of being committed or having to take medicine (394 of 1552 [22.8%]; weighted percentage) (Table 1). From 2011 to 2019, an increasing number of young adults reported not knowing where to go for services, fear of being committed or having to take medicine, having inadequate insurance coverage for treatment, fear of negative effect on jobs, and having concerns about confidentiality. No significant interaction effects were identified, suggesting that these time changes were consistent by young adults’ sociodemographic variables.

**Table 1.** Bivariate Analysis of Time Trends in Perceived Reasons for Not Seeking Mental Health Care Among Young US Adults With Untreated 12-Month Major Depression, 2011-2019

Reason	Participants, No. (%)^a^									OR (95% CI)
	2011 (n = 1177)	2012 (n = 1166)	2013 (n = 1173)	2014 (n = 941)	2015 (n = 1194)	2016 (n = 1244)	2017 (n = 1340)	2018 (n = 1399)	2019 (n = 1552)	
Could not afford cost	568 (51.1)	567 (56.7)	601 (54.6)	448 (48.3)	566 (50.0)	532 (43.5)	647 (53.6)	656 (48.6)	776 (54.7)	1.00 (0.96-1.03)
Did not know where to go for services	331 (24.9)	344 (27.5)	377 (28.9)	300 (30.8)	418 (35.3)	402 (28.6)	490 (37.9)	462 (34.6)	572 (37.8)	1.07 (1.03-1.11)^b^
Could handle problem without treatment	331 (25.2)	366 (29.3)	328 (28.6)	281 (30.8)	424 (32.8)	480 (36.6)	487 (31.6)	493 (32.3)	525 (30.9)	1.03 (0.99-1.07)
Fear of being committed or having to take medicine	185 (12.4)	229 (22.3)	214 (14.4)	208 (20.1)	269 (23.2)	295 (24.8)	325 (21.5)	297 (21.3)	394 (22.8)	1.06 (1.01-1.10)^c^
Did not have time	188 (14.2)	223 (19.0)	179 (17.8)	177 (19.9)	248 (19.8)	307 (25.0)	302 (22.4)	300 (20.2)	345 (18.8)	1.03 (0.99-1.08)
Fear of negative opinions of neighbors or community	148 (9.6)	153 (17.2)	201 (12.9)	167 (17.3)	217 (19.4)	231 (15.2)	233 (16.9)	250 (17.5)	302 (16.7)	1.04 (1.00-1.09)
Inadequate insurance coverage for treatment	62 (7.2)	83 (9.5)	78 (4.6)	101 (13.3)	117 (10.5)	156 (12.7)	170 (13.5)	186 (16.5)	216 (15.8)	1.13 (1.05-1.21)^b^
Fear of negative effect on jobs	93 (4.5)	102 (9.5)	107 (8.2)	107 (12.4)	148 (11.3)	168 (12.8)	188 (13.7)	191 (13.1)	209 (14.6)	1.11 (1.05-1.17)^b^
Concerns about confidentiality	90 (4.0)	169 (15.2)	143 (11.7)	101 (7.1)	207 (14.9)	214 (15.8)	206 (16.4)	226 (14.9)	234 (14.3)	1.09 (1.03-1.14)^c^
Did not think treatment would help	170 (13.2)	169 (9.7)	156 (17.7)	136 (16.2)	221 (17.6)	188 (13.0)	216 (15.5)	215 (12.9)	232 (13.7)	1.00 (0.95-1.05)
Fear of others finding out	117 (7.7)	156 (14.9)	127 (10.6)	123 (13.2)	169 (13.3)	168 (11.3)	195 (13.3)	203 (11.6)	204 (11.9)	1.01 (0.96-1.07)
No insurance coverage for mental health treatment	102 (8.0)	61 (6.5)	101 (9.7)	54 (4.5)	124 (9.9)	98 (7.1)	117 (11.5)	118 (9.3)	169 (11.3)	1.06 (1.00-1.13)
Did not think treatment was needed	96 (6.4)	124 (9.6)	117 (10.8)	98 (8.7)	148 (13.4)	182 (16.3)	145 (9.5)	163 (9.3)	181 (9.9)	1.02 (0.97-1.07)
Lack of transportation or inconvenient hours	46 (2.4)	61 (4.2)	68 (6.0)	44 (4.4)	79 (9.6)	84 (6.8)	107 (6.6)	61 (3.2)	105 (6.5)	1.04 (0.98-1.11)

Abbreviation: OR, odds ratio.

^a^ Unweighted numbers of participants and weighted percentages are reported for each survey year. For each bivariate regression model, the trend was considered statistically significant if the coefficient (ie, slope) of the year was statistically significant. From 2011 to 2019, between 97.4% and 99.3% of young adults who did not receive any treatment for depression reported at least 1 reason for not seeking mental health care in the National Survey on Drug Use and Health. No mathematical correction was made for multiple comparisons.

^b^ *P* ≤ .001.

^c^ *P* ≤ .01.

Compared with White participants, Hispanic and Asian participants were more likely to report not knowing where to go for services (Hispanic participants: adjusted odds ratio [AOR], 1.57 [95% CI, 1.21-2.03]; Asian participants: AOR, 2.63 [1.68-4.11]), whereas Native American participants were more likely to report having no insurance coverage (AOR, 3.44 [95% CI, 1.05-11.24]) (Table 2). Hispanic participants were also more likely than White participants to report fear of being found out by others (AOR, 1.95 [95% CI, 1.38-2.76]). Female participants were less concerned than male participants about negative opinions of neighbors or communities (AOR, 0.65 [95% CI, 0.51-0.83]) or about being found out by others (AOR, 0.72 [95% CI, 0.54-0.96]).

**Table 2.** Multivariate Differences in Perceived Reasons for Not Seeking Mental Health Care for Major Depression Among Young US Adults, 2011-2019^a^

Variable	No.	Did not know where to go for services		No insurance coverage for mental health treatment		Fear of others finding out		Fear of negative opinion of neighbors or community	
		%	AOR (95% CI)	%	AOR (95% CI)	%	AOR (95% CI)	%	AOR (95% CI)
Year	NA	NA	1.07 (1.03-1.11)^b^	NA	1.07 (1.00-1.14)^c^	NA	1.01 (0.95-1.06)	NA	1.04 (0.99-1.09)
Sex									
Male	4349	31.6	1 [Reference]	10.1	1 [Reference]	14.0	1 [Reference]	19.3	1 [Reference]
Female	6837	33.4	1.07 (0.87-1.30)	8.4	0.83 (0.60-1.14)	10.8	0.72 (0.54-0.96)^c^	14.3	0.65 (0.51-0.83)^b^
Age, y									
18-21	4412	36.4	1 [Reference]	7.2	1 [Reference]	16.7	1 [Reference]	24.5	1 [Reference]
22-25	6774	31.2	0.82 (0.68-1.00)	9.8	1.37 (1.00-1.89)	10.0	0.59 (0.45-0.78)^b^	12.5	0.41 (0.33-0.52)^b^
Race and ethnicity									
Asian or Native Hawaiian and Other Pacific Islander	622	53.5	2.63 (1.68-4.11)^b^	10.0	1.14 (0.54-2.39)	12.4	0.40 (0.25-0.62)	17.5	0.49 (0.38-0.63)
Black	1338	31.8	1.07(0.78-1.48)	6.0	0.63 (0.37-1.09)	8.7	0.80 (0.49-1.30)	16.1	1.06 (0.69-1.64)
Hispanic	2154	40.4	1.57 (1.21-2.03)^b^	8.8	1.01 (0.67-1.52)	19.3	1.95 (1.38-2.76)^b^	15.7	0.88 (0.63-1.21)
Native American	161	32.4	1.13 (0.49-2.60)	24.0	3.44 (1.05-11.24)^c^	18.1	1.16 (0.67-2.01)	12.6	1.06 (0.64-1.76)
White	6283	29.4	1 [Reference]	9.0	1 [Reference]	10.6	1 [Reference]	15.8	1 [Reference]
≥2 Races or ethnicities	628	33.0	1.13 (0.70-1.84)	14.7	1.72 (0.87-3.41)	11.4	1.09 (0.59-2.03)	22.0	1.49 (0.90-2.46)
Family income, $									
<20 000	3309	32.3	1 [Reference]	8.9	1 [Reference]	11.4	1 [Reference]	14.9	1 [Reference]
20 000-49 999	4015	34.4	1.13 (0.89-1.43)	9.8	1.05 (0.72-1.55)	11.2	1.04 (0.73-1.47)	16.4	1.26 (0.93-1.70)
50 000-74 999	1584	31.7	1.02 (0,75-1.38)	10.7	1.18 (0.71-1.96)	10.7	1.00 (0.66-1.52)	13.0	0.94 (0.63-1.41)
≥75 000	2278	31.3	0.93 (0.71-1.22)	6.8	0.72 (0.44-1.17)	14.5	1.34 (0.91-1.95)	18.7	1.31 (0.96-1.79)
Severe impairment									
No	4798	28.6	1 [Reference]	7.7	1 [Reference]	10.9	1 [Reference]	15.7	1 [Reference]
Yes	6363	34.7	1.33 (1.08-1.64)^d^	9.6	1.30 (0.91-1.88)	12.4	1.13 (0.83-1.53)	16.2	0.98 (0.75-1.28)

Abbreviations: AOR, adjusted odds ratio; NA, not applicable.

^a^ Unweighted numbers of participants and weighted percentages are presented. All variables listed were included in multivariable logistic regression models to assess whether participants reported specific reasons for not seeking mental health care for depression in the National Survey on Drug Use and Health.

^b^ *P* ≤ .001.

^c^ *P* ≤ .05.

^d^ *P* ≤ .01.

## Discussion

Although this study is limited by potential social desirability bias based on self-reports, cost was consistently the most prominent barrier to seeking depression treatment among young adults from 2011 to 2019. In addition, young adults increasingly reported inadequate insurance coverage for mental health treatment. Since its implementation in 2014, the Medicaid expansion has reduced the rate of uninsured individuals and improved access to care for adults with depression.^6^ Immediate policy actions are needed, therefore, to close the Medicaid coverage gap, especially for Native American individuals. More outreach campaigns are also warranted to increase young adults’ awareness of local mental health services, particularly among Hispanic and Asian communities. Last, destigmatizing mental health treatment should be prioritized among young adults, with gender-specific engagement interventions for men.
